# Controlled dual release of dihydrotestosterone and flutamide from polycaprolactone electrospun scaffolds accelerate burn wound healing

**DOI:** 10.1096/fj.202101803R

**Published:** 2022-04-08

**Authors:** Huaikai Shi, Kevin H.‐Y. Tsai, Duncan Ma, Xiaosuo Wang, Reena Desai, Roxanne J. Parungao, Nicholas J. Hunt, Yuen Yee Cheng, Hao Zhang, Ye Xu, Ulla Simanainen, Qian Tan, Mark S. Cooper, David J. Handelsman, Peter K. Maitz, Yiwei Wang

**Affiliations:** ^1^ Burns Research and Reconstructive Surgery, ANZAC Research Institute, Concord Hospital University of Sydney Sydney Australia; ^2^ Asbestos Disease Research Institute Concord Hospital Sydney Australia; ^3^ Adrenal Steroids Laboratory, ANZAC Research Institute, Concord Hospital University of Sydney Sydney Australia; ^4^ Bosch Mass Spectrometry Facility University of Sydney Sydney Australia; ^5^ Department of Andrology, ANZAC Research Institute, Concord Hospital University of Sydney Sydney Australia; ^6^ Biogerontology Group, ANZAC Research Institute, Concord Hospital University of Sydney Sydney Australia; ^7^ Sydney Nano Institute University of Sydney Sydney Australia; ^8^ Charles Perkins Centre University of Sydney Sydney Australia; ^9^ Department of Burns and Plastic Surgery, Nanjing Drum Tower Hospital The Affiliated Hospital of Nanjing University Medical School Nanjing China; ^10^ Burns Unit Concord Repatriation General Hospital Concord Australia; ^11^ Jiangsu Provincial Engineering Research Centre of TCM External Medication Development and Application Nanjing University of Chinese Medicine Nanjing China

**Keywords:** androgens, burn injury, controlled drug delivery, flutamine, PCL scaffold, wound healing

## Abstract

Wound healing is a complex process involving multiple independent and overlapping sequential physiological mechanisms. In addition to cutaneous injury, a severe burn stimulates physiological derangements that induce a systemic hypermetabolic response resulting in impaired wound healing. Topical application of the anti‐androgen drug, flutamide accelerates cutaneous wound healing, whereas paradoxically systemic dihydrotestosterone (DHT) improves burn wound healing. We developed and characterized a PCL scaffold that is capable of controlled release of androgen (DHT) and anti‐androgen (F) individually or together. This study aims to investigate whether local modification of androgen actions has an impact on burn injury wound healing. In a full‐thickness burn wound healing, mouse model, DHT/F‐scaffold showed a significantly faster wound healing compared with F‐scaffold or DHT‐scaffold. Histology analysis confirmed that DHT/F‐scaffold exhibited higher re‐epithelization, cell proliferation, angiogenesis, and collagen deposition. Dual release of DHT and F from PCL scaffolds promoted cell proliferation of human keratinocytes and alters the keratinocyte cell cycle. Lastly, no adverse effects on androgen‐dependent organs, spleen and liver were observed. In conclusion, we demonstrated DHT plus F load PCL scaffolds accelerated burn wound healing when loading alone did not. These findings point to a complex role of androgens in burn wound healing and open novel therapeutic avenues for treating severe burn patients.

**Abbreviations**
DHT: dihydrotestosterone
DF: dual drug
F: flutamide
HaCaT: human keratinocytes
PCNA: proliferating cell nuclear antigen
PCL: polycaprolactone

## 1 INTRODUCTION

Burn injuries are among the most physically and psychologically debilitating injuries in people of all ages. In addition to skin injury, severe burns stimulate physiological derangements that induce a systemic hypermetabolic response. If not corrected in time, the hypermetabolic response can lead to weight reduction, loss of lean body mass, impaired wound healing, and sepsis.^1^, ^2^ These syndromes are associated with delayed recovery and prolonged hospital admission, increased morbidity, and mortality.^3^

Both cutaneous injury (non‐burn) and burn injuries immediately trigger the local wound healing process that involves four overlapping phases of hemostasis, inflammation, cell recruitment, and matrix remodeling. Rodent models have established that aromatize androgens like testosterone inhibit and prolong cutaneous wound healing in male mice,^4^, ^5^, ^6^, ^7^ an effect reversed by treatment with topical flutamide, an androgen antagonist.^8^, ^9^ However, in contrast, the more potent, pure non‐aromatizable androgen dihydrotestosterone (DHT) when administered systemically improved burn wound healing with earlier cell proliferation and collagen deposition.^10^ These novel findings point to a previously unrecognized, paradoxical, and context‐dependent role of androgens in wound healing that differs between burn and cutaneous injuries. The paradoxical, beneficial effects of systemic pure androgen administration on local wound healing raise the question of whether androgen receptor (AR)‐mediated androgen actions may have an overlooked positive effect in local cutaneous healing of burn injury. Understanding the local androgen action may lead to a novel therapeutic avenue in treating severe burn injury exploiting the potential to modulate local androgen actions.

Current topical treatments using an aqueous‐based cream, petroleum jelly or oil‐based vehicle have limitations due to their uncontrolled drug delivery including an initial burst release, which can lead to unwanted high circulating drug levels.^11^ For instance, although topical cutaneous treatments with anti‐androgens, such as flutamide, are reported to accelerate wound healing, oral flutamide treatment has significant potential adverse effects, including fatal hepatoxicity, as well as sexual dysfunction, anemia, and reduced muscle and bone mass.^12^ Furthermore, in full‐thickness severe burn injuries, skin is severely damaged so that topical treatments via wound dressing materials are necessary to protect the wound bed, and provide a moist environment to facilitate healing, but cannot replace lost tissues.^11^, ^13^ Therefore, to aid tissue regeneration as well as controlled delivery of anti‐androgens, biomaterials scaffolds are expected to achieve optimal, faster wound healing.

Our lab has recently developed a soft, non‐woven electrospun collagen‐PCL scaffold that can control the release of anti‐androgens such as hydroxyflutamide (HF). Application of the HF scaffold has demonstrated accelerated cutaneous burn wound healing compared with the blank collagen‐PCL scaffold by day 14, with wound biopsies showing multiple layers of fibroblasts adhering to the scaffold.^8^, ^14^ In the present study, we firstly developed and characterized a PCL scaffold for controlled delivery of androgen (DHT), the anti‐androgen flutamide (F) individually or the combination of DHT and F. Following which we investigated using in vitro examination and in vivo assessments on cutaneous burn injured mice. Secondly, we investigate whether modulation of local androgen actions could enhance burn injury wound healing with a focus on cell recruitment, proliferation, and collagen deposition. In addition, the systemic effects and safety profiles of scaffold treatment were investigated.

## 2 MATERIALS AND METHODS

### 2.1 Fabrication of electrospun polycaprolactone scaffolds

DHT, flutamide (F), and DHT/F mixed (50:50) powder (Sigma‐Aldrich) was dissolved in 5% polycaprolactone (PCL) (w/v) solution in 1,1,1,3,3,3‐hexafluoro‐2‐propanol (HFP) (Sigma Aldrich) to produce 5 mg/ml of DHT‐PCL, F‐PCL or DHT+F (DF)‐PCL solution, respectively. The blank‐PCL solution containing 5% PCL was used as a control. Over electrospinning, 0.8 ml of DHT‐PCL, F‐PCL, DF‐PCL, or blank‐PCL solution was loaded into a 1‐ml syringe. The flow rate was set at 1 ml/h (SP 100iZ, World Precision Instruments Inc., FL, USA), air gap distance was set at 20 cm, and an electric potential (+10V/−8V) was applied in accordance with previous studies.^8^

### 2.2 Surface analysis of drug loaded electrospun polycaprolactone scaffolds

DHT‐PCL, F‐PCL, DF‐PCL, or blank scaffolds were mounted on aluminum sample stubs and sputter‐coated with platinum using an auto coater at 45 nm (JFC‐1600 Auto Fine Coater, JEOL Ltd., Tokyo, Japan) prior to examination by scanning electron microscopy (SEM) (JEOL JSM‐6380, JEOL Ltd., Tokyo, Japan) at a voltage of 15 kV. The surface morphology of DHT‐PCL, F‐PCL, DF‐PCL, and blank‐PCL scaffolds before and after drug delivery on day 31 were both examined.

### 2.3 Drug release profile

#### 2.3.1 In vitro release of DHT and F from PCL scaffolds

Samples of electrospun PCL scaffolds (weight of 5 mg, *n* = 3) containing DHT, F, or DF at a concentration of 5 mg/ml were immersed in 1 ml phosphate‐buffered saline (PBS) (pH 7.4) at 37°C for 31 days, respectively. The medium was collected and completely replaced by fresh PBS at 1‐day intervals. The amount of F released into PBS was analyzed by UV spectrophotometer (Implen®, München, Germany) at a wavelength of 230 nm compared with a standard curve of a serial dilution of F from 6.25 to 125 µg/ml in PBS. The amount of DHT release into PBS was analyzed by liquid chromatography‐mass spectrometry (Shimadzu Nexera UHPLC) linked to an API‐5000 triple‐quadrupole mass spectrometer (Applied Biosystems/MDS SCIEX, Foster City, Ontario, Canada)^15^ compared with a standard curve of serial dilution from 3 to 50 ng/ml.

#### 2.3.2 In vivo validation of controlled drug delivery from PCL scaffolds

##### Orchidectomy

To confirm if delivery of androgens or anti‐androgens from scaffolds released to local, systemic circulation or both, orchiectomy was used to remove endogenous testosterone and eliminate the reflex stimulation of endogenous androgens, respectively. Twelve‐week‐old male Balb/c mice (Animal Resource Centre, Murdoch, WA, Australia) (*n* = 24, 3 mice per time point) were castrated 7 days prior to burn injury. For orchiectomy, all animals were under ketamine/xylazine anesthesia, a small incision <1 cm was made in the scrotal skin to exteriorize the testes. The testicular vessels were sutured (4/0 silk) and the testes were removed without blood loss prior to closure with 1–2 sutures (4/0 silk, Total Patient Care, NSW, Sydney, Australia). Analgesia by administration of Carprofen (5 mg/kg, Norbrook®, Newry) was provided for the first 2 days, and all animals were closely monitored daily. The surgical procedures were approved by the Sydney Local Health District Welfare Committee (Protocol No. 2018/020) under the Australian NHMRC Guidelines for animal experimentation.

##### Detection of serum DHT concentration by LC‐MS/MS

Stock solutions of T and DHT were prepared by weighing the powder on an analytical digital balance, followed by dissolution in methanol. Serial concentrations for both T and DHT (16, 8, 4, 1.6, 0.8, 0.4, 0.2, 0.1, 0.05, 0.025, and 0.01 ng/ml) were prepared by diluting the stock solution with 4% BSA (w/v) in PBS for generating the calibration curve. Quality control (QC) samples were prepared in charcoal‐stripped plasma at low, medium, and high levels. Internal standard (IS) solutions (5 ng/ml d3‐T and d3‐DHT, NMI, Sydney, Australia) were prepared in methanol and diluted with 20% (v/v) methanol in water. DHT‐PCL scaffold medium used for in vitro drug delivery was collected and filtered by a 0.2‐μm PES filter (to remove PBS) before being analyzed by LC‐MS/MS.^15^

For samples collected from in vivo studies, aliquots (200 µl) of thawed serum, standards or QC were transferred into 5‐ml glass tubes. 50 µl of deuterated steroid ISs were added to 50% (v/v) methanol. All samples were vortexed at 4°C for 15 min, while 1 ml of methyl tert‐butyl ether was added prior to vigorously mixing (1 min) to extract steroids into the organic solvent (upper layer). All test tubes were then covered with parafilm and allowed to phase separate at 4°C for 1 h. After 1 h at 4°C, the tubes were placed in a −80°C freezer for 30 min to freeze the lower aqueous layer. The upper organic layer was then decanted into clean glass tubes, and the solvent was allowed to evaporate in a fume hood overnight. For steroid analysis, the dried samples were re‐suspended in 75 µl of 20% (v/v) methanol. Tubes were well mixed for 1 min, and the entire volume was then transferred into a 96‐well plate. A 50 µl aliquot was injected onto the column for LC‐MS/MS analysis.^15^ The retention time for T and DHT was 3.28 min and 4.19 min, respectively (Figure S1).

### 2.4 In vivo severe burn injury mice model

#### 2.4.1 Full‐thickness severe burn injury wound healing

Male BALB/C mice (*n* = 24 from Animal Resource Centre, Murdoch, WA, Australia) were randomly assigned to one of the following groups (*n* = 6/group): control (blank), androgen treatment (5 mg/ml, DHT scaffold), anti‐androgen treatment (5 mg/ml, F scaffold), or dual drug treatment (5 mg/ml, DHT and F mixed scaffold).

#### 2.4.2 Histology, immunocytochemistry, and image analysis

Wound, liver, and spleen tissues were embedded in paraffin. Multiple 5 μm sections were stained with hematoxylin and eosin (H&E) (Sigma‐Aldrich) for general histological analysis; F4/80 (1:100, Abcam) for inflammation, proliferating cell nuclear antigen (PCNA) (1:1000, Abcam) for proliferation; CD146 (1:100, Abcam) for anagenesis and Picro‐Sirius red staining with polarized imaging for collagen deposition.

#### 2.4.3 Liver toxicity test

Biochemical liver toxicity was assessed by serum aspartate aminotransferase (AST) and alanine aminotransferase (ALT) concentrations using commercial assays (MAK055 and MAK052, Sigma‐Aldrich) performed as per kit instructions.

### 2.5 In vitro keratinocytes culture studies

#### 2.5.1 In vitro drug treatment

Approximately 5 mg DHT‐PCL, F‐PCL or DF‐PCL scaffolds were placed in a 24‐Well Millicell® hanging cell culture insert (1.0 μm, Millipore, Sigma‐Aldrich), then immersed into culture medium with immortalized human keratinocytes (HaCaT), which were kindly provided by Burns Unit (Concord Hospital, NSW, Sydney, Australia). The culture plate was shaken daily to ensure the release and even distribution of the drugs in the cell culture medium.

#### 2.5.2 Wound scratch assay

HaCaT cell migration was measured using a scratch (wound‐healing) assay. Briefly, cells were plated in 24‐well plates and 24 h post‐seeding, 10 µg/ml Mitomycin C (Sigma‐Aldrich) was added to stop cell division; at the same time, a scratch was made using a plastic pipette tip. At 24, 48 h post scratch, microscopic imaging was taken with a 20× objective (Leica). Each experiment group was performed in triplicate.

#### 2.5.3 Cell proliferation assay

AlamarBlue® cell death assays (proliferation assay) were conducted for human keratinocytes. Briefly, cells were plated in 24‐well culture plates at 20 000 cells in 200 μl medium per well. After 24 h, AlamarBlue®, 20 μl (50 ml PBS containing reagents 0.075 g of resazurin, 0.0125 g of methylene blue, 0.1655 g of potassium hexacyanoferrate (III), 0.211 g of potassium hexacyanoferrate (II) trihydrate, filter‐sterilized, and stored at 4°C in the dark) was added and incubated for 4 h at 37°C. Fluorescence intensity was measured at 590 nm with 544 nm excitation using a FLUOstar Optima (BMG LabTech, Ortenberg, Germany). Fluorescence intensity was presented as a percentage of the intensity of control cells. Experiments were performed three times with three replicates each time, except for experiments involving slow‐growing non‐cancer primary fibroblasts that were performed four times with two or three replicates.

#### 2.5.4 Cell cycle analysis

After scaffold treatment for 48 h, cells were harvested and washed three times with PBS. Cells were either fixed with 70% ethanol for cell cycle analysis. For cell cycle analysis, the fixing solution was removed, and cells were treated with 0.01% RNase (10 mg/ml, Sigma‐Aldrich), 0.05% propidium iodide (PI)(Sigma‐Aldrich) in PBS for 30 min at 37°C in the dark. The cell cycle distribution was determined on a Cytoflex (Beckman Coulter, Miami Lakes, FL) within 30 min. The flow cytometer was calibrated using 6‐ and 8‐peak fluorescent bead mixtures provided by the manufacturer according to their instructions (Accuri, Ann Arbor, MI, USA). The flow cytometer was routinely operated at the slow flow rate setting (14 μl sample/min), and data acquisition for a single sample typically occupied 3–5 min. For each sample, 10 000 events of single cells were counted, and the cell cycle was analyzed using FlowJo software (Ashland, OR, USA).

### 2.6 Statistical analysis

Repeated measurement analysis of variance (ANOVA) was used for all continuous variables including wound healing rate to correct between‐animal bias caused by multiple observations from each animal. The data are presented as mean ± SEM, and significant differences were determined by two‐way ANOVA and paired *t*‐test with *p* ≤ .05 accepted for statistical significance. *N* = 3–6 per group per time point *p < .05, **p < .01, ***p < .001.

## 3 RESULTS

### 3.1 Production of drug‐loaded scaffold and controlled drug delivery in vitro

**FIGURE 1:**
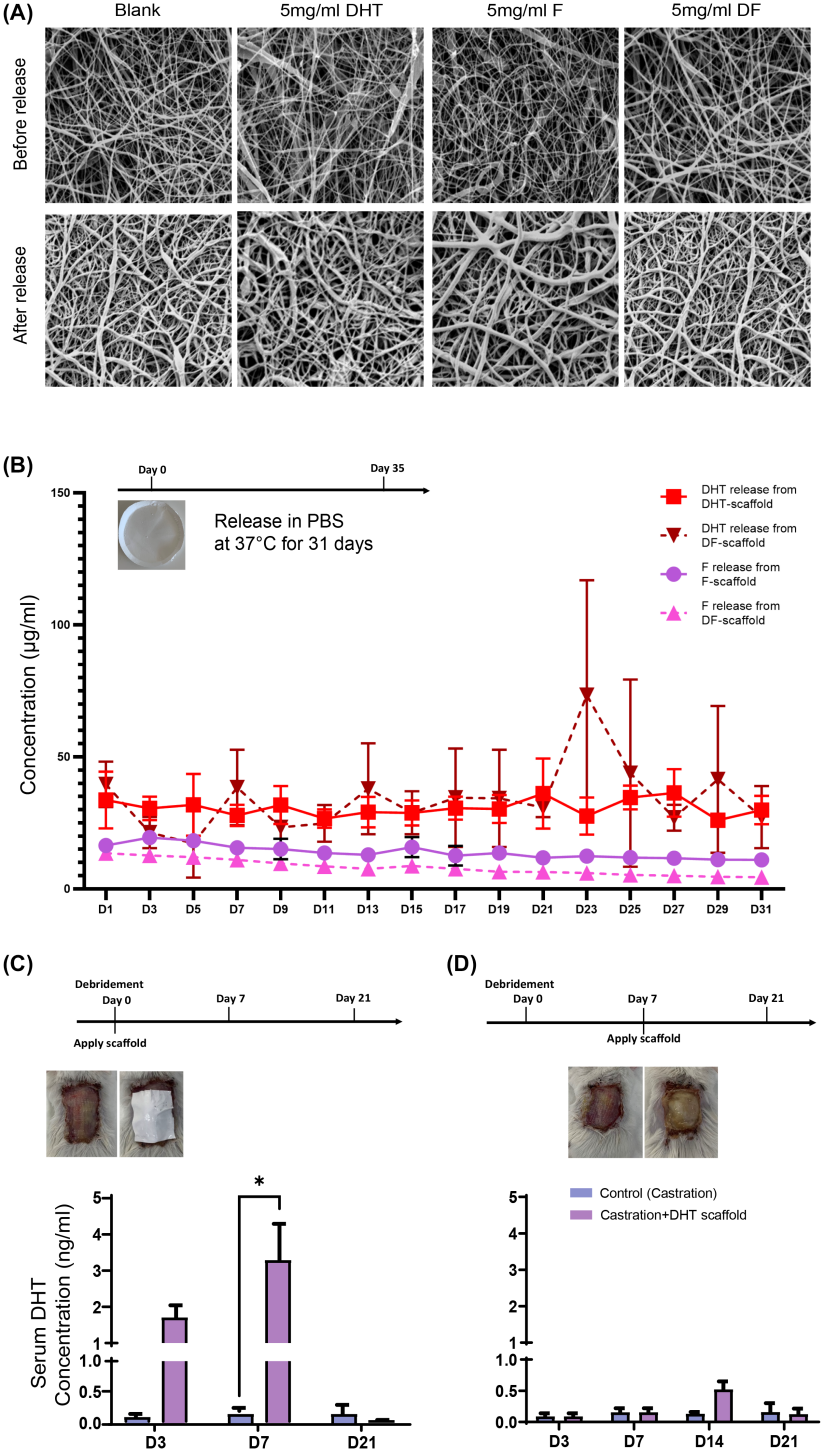
(A) SEM analysis of drug loaded electrospun PCL scaffolds before and after drug delivery. (B) both DHT and F were released constantly into PBS at 37°C from the PCL scaffolds without initial burst release over 31 days. Data are shown as mean ± SEM; *N* = 3. (C) Burst release of DHT into the circulation was detected on day 7 by LC‐MS when PCL scaffolds were applied on day 0 post‐burn injury. (D) Local DHT delivery was achieved with PCL scaffolds applied on day 7 post‐burn injury. *N* = 6 per group per time point, *p < .05

DHT‐PCL, F‐PCL, DF‐PCL, or blank‐PCL scaffolds were all produced successfully by electrospinning. SEM analysis of drug‐loaded PCL scaffolds showed that all scaffolds had a soft and porous surface morphology with the majority of fiber areas circular and thread‐like, whereas minimal fibers were occasional flat, ribbon‐like shapes. No drug particles were seen on the surface or extruded from PCL fibers, indicating that dispersed DHT and F were well coated by PCL polymers during formation (Figure 1A).

Approximately 33.7 ± 10.8 µg of DHT from DHT scaffold and 16.5 ± 2.0 µg of F from F scaffold were released into 1 ml of PBS at 37°C in the first 24 h. In DF mixed scaffold, approximately 26.2 ± 11.6 µg of DHT and 13.6 ± 0.4 µg of F were released, respectively, which is very close to single drug‐loaded scaffolds of DHT or F (Figure 1B). Thereafter, 25–38 µg of DHT and 15–20 µg of F were constantly released every 2 days over 31 days (Figure 1B). After drug delivery from scaffolds, all scaffolds were found to maintain well their original shape with no obvious cracks, surface depression, or dispersed drug noted (Figure 1A). There was no initial burst release of drugs detected in the DHT, F, or DF scaffold into saline.

### 3.2 Validation of controlled drug delivery in vivo

#### 3.2.1 Orchidectomy

Orchidectomy was used to delete endogenous androgens prior to DHT scaffold treatment to accurately measure the in vivo released amount of DHT. After orchidectomy, serum testosterone level dropped from 1 ng/ml to a non‐detectable level (<0.01 ng/ml) and DHT was reduced from 0.11 to 0.04 ng/ml in castrated mice (Figure S2).

#### 3.2.2 Burst release of androgen in circulation after post scaffold's application

After orchidectomy, electrospun DHT scaffolds were firstly applied after debridement, resulting in an increase in circulating DHT concentration 3 and 7 days after the application of the DHT scaffold. Serum DHT concentration at 7 days post‐administration, increased in castrated mice from 0.03 to 3.5 ng/ml in mice receiving DHT scaffolds (Figure 1C).

#### 3.2.3 Controlled local drug delivery via applying scaffold on day 7 post‐debridement

To overcome burst release of DHT from scaffolds into the systemic circulation (Figure 1C), the DHT scaffold was then applied to the wound area after day 7 post‐debridement, once the granulation layer had formed. Thereafter, the serum DHT concentration was measured on day 3 (before scaffold application), 7, 14, and 21 (0, 7, and 14 days after scaffold application) and compared with the castrated control animals (Figure 1D). LCMS measurements showed that the DHT concentration remained fairly low in both experimental and control groups with no significant differences between each group up to 21 days apart from a small increase in serum DHT concentration 7 days after receiving DHT scaffold in castrated mice (0.4 vs. 0.1 ng/ml in controls) (Figure 1D). The serum DHT concentration was found significantly lower compared with those animals having scaffold on day 0 after debridement (3.5 ng/ml, *p* < .05). This observation indicated that when scaffolds were applied on day 7 post‐debridement with the regeneration of granulation tissue, forming a skin barrier that could reduce DHT released into circulation. Therefore, in the following in vivo studies, all drug‐loaded scaffolds, including DHT, F, and DF were applied on day 7 post‐debridement. These experiments demonstrated the suitability of PCL scaffolds as a local drug‐delivering device to investigate the local effects of androgen as well as to minimise the adverse systemic effects with steroid treatments.

### 3.3 Burn injury wound healing was significantly accelerated in F and DF‐PCL scaffold‐treated mice

**FIGURE 2:**
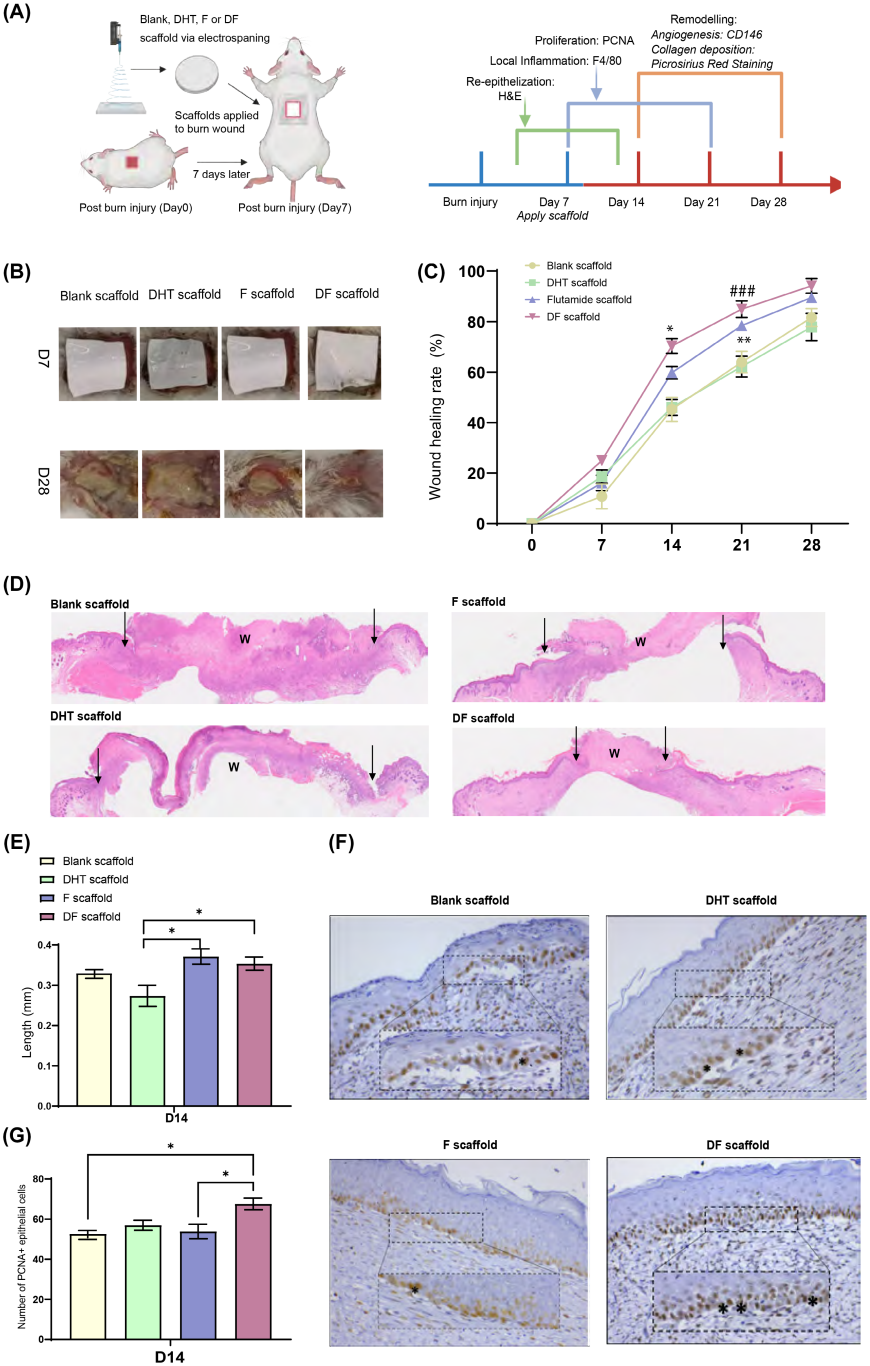
(A) Scaffold treatments were applied to the burn wound and assessments were performed according to the wound healing process. (B) DF scaffold displayed fastest wound recovery after scaffold treatments. (C) F and DF mixed scaffold treatments promote burn injury wound healing with the highest percentage of wound healed found in DF mixed scaffold at days 14 and 21. *DF versus F, *p* < .05, **F versus DHT, *p* < .01, ^##^DF versus DHT, *p* < .001, *N* = 6 per group per time point, error bar = SEM. (D) DF scaffold‐treated wound displayed smallest remaining wound area (black arrow) by H&E at day 14. (E) DF and F scaffold‐treated wound demonstrated significant higher length of re‐epithelization compared with DHT scaffold. *p < .05, *N* = 6 per group per time point, error bar = SEM. (F) and (G) DF scaffold‐treated wound demonstrated significant higher number of PCNA+ epithelial cells compared with F scaffold and blank scaffold‐treated wound. Black stars indicate the PCNA+ epithelial cells. *p < .05, *N* = 6 per group per time point, error bar = SEM

We explored the local effect of androgen actions on in vivo burn injury wound healing through the application of androgen (DHT), anti‐androgen (F), or dual drug (DF) PCL scaffolds, respectively (Figure 2A). Animals that received F scaffolds showed a significantly faster wound healing rate when compared with the mice treated with either DHT or blank scaffolds (Figure 2B,C). On day 14 and day 21, wounds treated with F scaffolds healed by approximately 59.8% ± 2.5% and 78.4% ± 1.5%, respectively, compared with 46.0% ± 3.1% and 59.8% ± 2.5% of the healed wounds in the group of DHT scaffolds (Figure 2B, Table 1). Additionally, the burn injury wound healing was further promoted in the group receiving DF‐PCL scaffolds. Approximately 70.4% ± 2.9% wound healed on day 14 and 85.0% ± 3.3% on day 21 compared with the mice having F scaffolds (Figure 2B,C, Table 1). On day 28, approximately 76%–80% of the wound healed in mice with DHT or blank scaffolds, whereas mice with DF scaffold demonstrated full healed wound at approximately 95% ± 2.7%.

**TABLE 1:** Wound healing rate comparison post scaffold treatments in burn injured mice

	Day 14	Day 21
Blank scaffold	44.5% ± 2.2%	59.3% ± 4.8%
DHT scaffold	46.0% ± 3.1%	59.8% ± 2.5%
F scaffold	59.8% ± 2.5%	78.4% ± 1.5%
DF scaffold	70.4% ± 2.9%	85.0% ± 3.3%

Note

DF scaffold‐treated animals showed significantly higher percentage of healed wounds at both day 14 and day 21.

Histological analysis of H&E staining revealed significantly reduced wound area and faster re‐epithelialization in DF‐PCL and F‐PCL groups at day 14. In DF‐PCL and F‐PCL scaffold‐treated mice, re‐epithelization, an early indication of wound healing was found to reach approximately 0.35 mm and 0.37 mm, respectively, compared with 0.27 mm in animals receiving DHT‐PCL scaffolds (Figure 2D,E). To determine if faster re‐epithelization was because of faster cell proliferation of keratinocytes in the epidermis, skin samples were collected on day 14 for PCNA staining. At day 14, DF‐PCL scaffold‐treated mice displayed a greater percentage of PCNA positive cells in the migrating epidermis compared with that found in F‐PCL or blank scaffold‐treated mice (67.6% ± 2.9% vs. 53.8% ± 3.6% in F and 52.6% ± 3.5% in blank control; Figure 2F,G).

**FIGURE 3:**
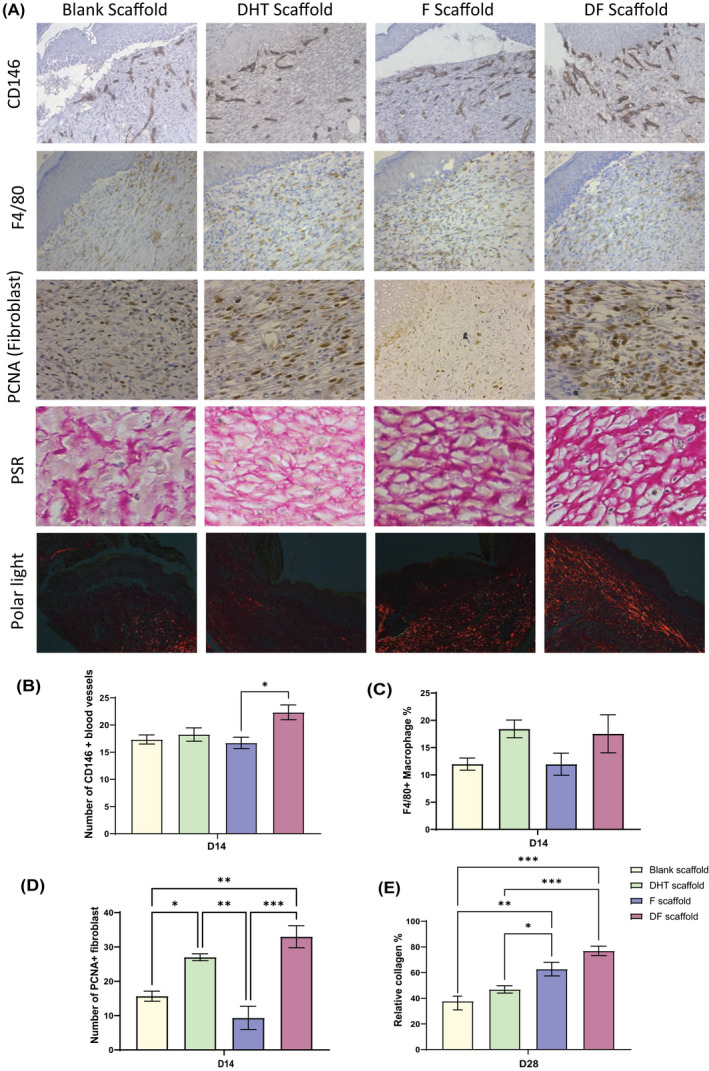
(A) IHC staining images of CD146, F4/80, PCNA at day 14 and PSR/PSR polarization light microscopy at day 28 (B) DF scaffold‐treated wound demonstrated significant higher number of CD146+ blood vessels compared with F scaffold, indicating enhanced angiogenesis at day 14. *N* = 6 per group, *p > .05, error bar = SEM (C) no significant difference was found for F4/80+ macrophage at wound site in all scaffold‐treated animals. *N* = 6 per group, error bar = SEM (D) DF scaffold‐treated mice demonstrated a significantly higher number of PCNA+ fibroblast at day 14, *N* = 6 per group, *p < .05, **p < .01, ***p < .001, error bar = SEM (E) collagen deposition also enhanced in DF scaffold‐treated wound at day 28. *p < .05, **p < .01, ***p < .001, *N* = 6 per group per time point

We then compared the expression of CD146, a biomarker on vascular endothelium, which is involved in angiogenesis and F4/80 for positive macrophage infiltrated into the wound site post‐scaffold treatment (Figure 3A). The DF scaffold‐treated mice revealed the highest number of CD146‐positive stained blood vessels (avg. 22 blood vessels per 30 mm^2^) compared with an average of 16 blood vessels per 30 mm^2^ in other experimental groups (Figure 3B). The number of F4/80 positive macrophage at the wound area is similar among all groups with a slight increase in both DHT and DF scaffold‐treated mice at day 14 (Figure 3C). Picrosirius red polarized light microscopy revealed an enhanced collagen deposition in DF scaffold‐treated mice on day 28 (Figure 3A). Collagen density in the wound area of DF scaffold‐treated mice was measured at 76.9% ± 7.4% on day 28, which is significantly higher than 46.9% ± 7.6% in DHT and 37.7% ± 2.9% in blank scaffold‐treated mice (Figure 3E). The collagen fibers in DF group are significantly denser and organized compared with others (Figure 3A). Because the fibroblast plays an important role in collagen synthesis, we further assessed the proliferating fibroblast at the wound site (Figure 3A). DF scaffold‐treated mice showed a significantly higher number of PCNA‐positive fibroblasts compared with that of F or blank scaffold‐treated animals (avg. 33 cells per 30 mm^2^ in DF scaffold vs. 9.33 in F and 15.67 in blank scaffold) (Figure 3D).

### 3.4 Drug‐loaded PCL scaffolds had no systemic adverse effects

**FIGURE 4:**
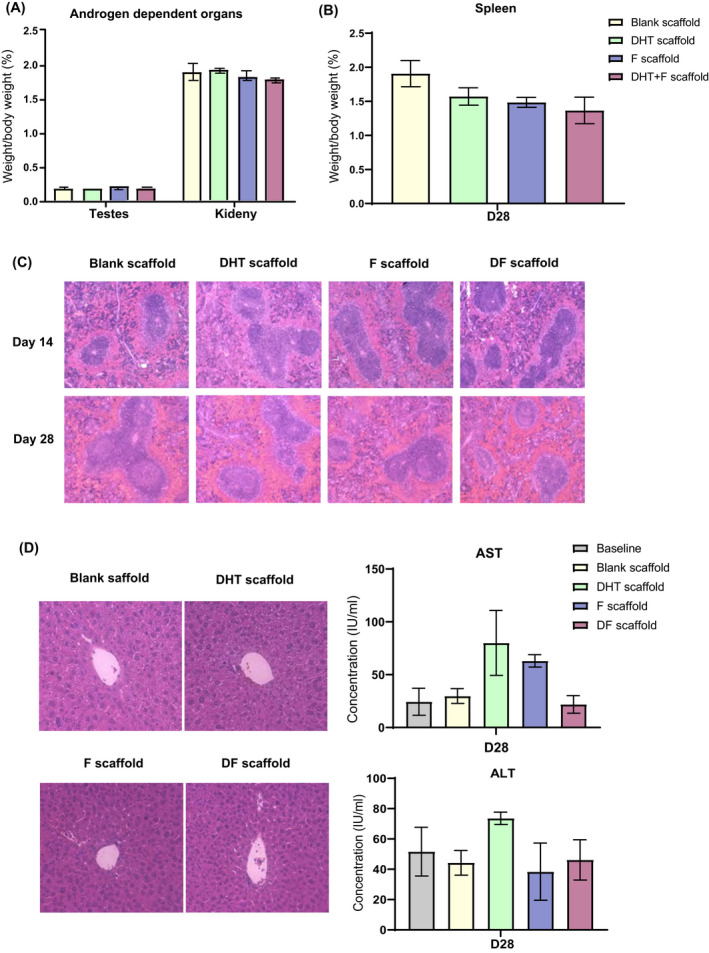
No significant change in the weight (w/w) of (A) androgen‐dependent organ testes and kidney and (B) spleen was observed at the endpoint of scaffold treatments (day 28) compared with the blank control. *N* = 6 per group, error bar = SEM (C). H&E staining demonstrated normal spleen histology with distinct white pulp and red pulp post scaffold treatments at day 14 and 28. (D) No histological changes were observed in liver samples after scaffold treatments. Furthermore, no differences in serum AST or ALT concentrations were observed compared with untreated controls. *N* = 6 per group, error bar = SEM

The systemic effects of different drug‐loaded scaffolds were studied on androgen‐dependent organs (kidney, testes) at day 28 post‐treatment. Both kidney and testes showed similar weight (w/w) and histology on day 28 between all experimental groups compared with blank scaffold‐treated mice (Figure 4A). This finding indicated that local modification of androgen via controlled delivery of DHT or F from scaffold had no systemic effect on androgen‐dependent organs over 28 days. Additionally, no significant differences were found in the weight (w/w) of the spleen in all animals over 28 days. Histologic analysis demonstrated distinct white pulp and red pulp distribution, and normal immune cell composition in both the white pulp and red pulp. There were no significant differences between the immune cell composition in the mice spleen at day 14 and day 28, respectively, in all three experimental groups (Figure 4B,C) and no difference compared with the scaffold‐treated control. This result further supported that these scaffold treatments were not associated with altered splenic structure and function. No differences in serum AST or ALT concentrations were observed between treatment groups and untreated controls (Figure 4D). The normal liver enzyme findings were further supported by the normal histologic assessment of the mice liver. There were no changes in hepatocytes lobular architecture post‐scaffold treatment, and no hepatocyte necrosis or significant portal inflammatory infiltrate was observed. A minor increase in portal infiltration of immune cells mainly lymphocytes, were observed in all three experimental groups, but no acute or chronic hepatitis, or liver fibrosis present. Overall, there are no observes changes in androgen‐dependent organ, liver and spleen with scaffold drug delivery.

### 3.5 DF‐PCL scaffolds significantly enhanced human keratinocyte cell line (HaCaT) proliferation but not cell migration

**FIGURE 5:**
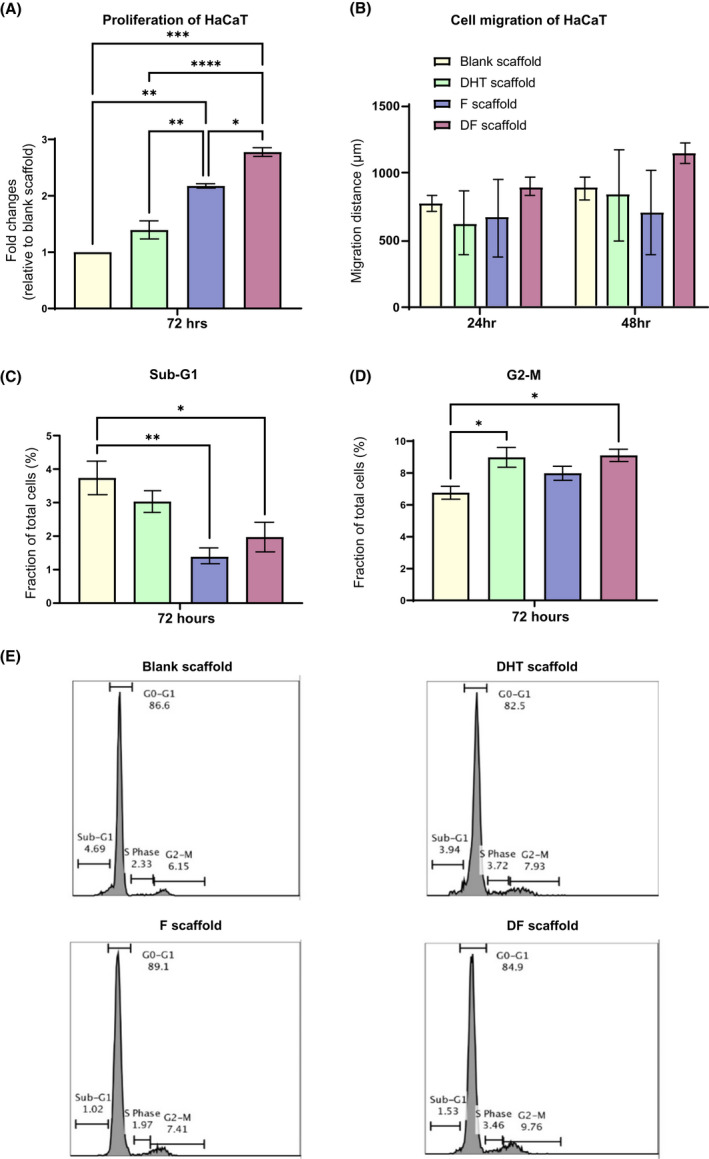
(A) Proliferation of HaCaT under scaffold treatments, DF scaffold treatment significantly enhanced HaCaT proliferation compared with that of DHT and F treatments. *N* = 3 per group, *p < .05, **p < .01, ***p < .001, ****p < .0001 (B) PCL scaffold treatments showed similar HaCaT migration distance post 24 and 48 h. *N* = 3 per group per time points (C) and (D) HaCaT cell cycle analysis revealed that a synergistic positive effect of F (reduce Sub‐G1) and DHT (extent G2‐M), correlated to the significant enhanced HaCaT proliferation observed from DF scaffold treatment. *N* = 3 per group, *p < .05, **p < .01, ***p < .001. (E) Flow cytometry analysis of HaCaT cell cycle

To investigate drug effects on keratinocytes proliferation, a steroid‐free CS‐FBS culture medium was used, and no differences in cell proliferation were noted for steroid‐free CS‐FBS or normal FBS. After 72 h of drug treatments, cell proliferation of HaCaT was significantly increased with either F‐PCL or DF‐PCL scaffolds compared with blank or DHT‐PCL scaffolds. Cell numbers of keratinocytes in the F‐PCL scaffold‐treated increased by 1.2‐fold compared with blank scaffold and reduced to 0.8‐fold with DHT‐PCL scaffold (Figure 5A). The DHT plus F mixed scaffold demonstrated a synergistic effect, further enhanced cell proliferation by 1.7‐fold compared with F‐PCL scaffolds (Figure 5A), this could be the reason for in vivo faster wound healing and re‐epithelization. In the in vitro scratch assay, all scaffold treatments exhibited a similar cell migration rate of HaCaT at both 24 and 48 h (Figure 5B).

### 3.6 DF‐PCL scaffolds enhance keratinocyte cell proliferation by altering cell cycle

Whether F‐PCL and DF‐PCL enhanced keratinocyte cell proliferation due to alteration of the cell cycle was further examined. The cell cycle profile of the G0‐G1 and S phase appeared to be similar when keratinocytes were treated with DHT, F, or DF‐PCL scaffolds. However, the fraction of total cell in the sub‐G1 phase was significantly reduced in F or DF‐PCL (1.39% ± 0.49% in F‐PCL scaffold and 1.98 ± 0.88 in DF‐PCL scaffold) compared with DHT‐PCL or blank control scaffold (3.03 ± 0.68 in DHT‐PCL scaffold and 3.71 ± 0.98 in blank scaffold) after 72 h (Figure 5C). Upon G2‐M phase, the fraction of total cells was significantly increased in DHT or DF‐PCL scaffolds recording 8.81% ± 1.47% for DHT‐PCL scaffolds and 9.11% ± 0.76% for DF‐PCL scaffold, respectively, while blank control was measured at only 6.76% ± 0.81% (Figure 5C,D). These findings support that the DF‐PCL scaffold had synergistic effects on keratinocyte proliferation as the dual release of DHT and F was capable to reduce the sub‐G1 phase (cell growth) by anti‐androgen F and promoted G2‐M phase (cell mitosis) phage by androgen DHT (Figure 5E).

## 4 DISCUSSION

In the present study, the application of biomaterial scaffolds with controlled delivery of a pure potent non‐aromatizable androgen (DHT) with or without an anti‐androgen (flutamide) was investigated with a focus on androgen effects on local burn wound healing. Although local pharmacological AR blockade by a topical flutamide cream is reported to promote cutaneous wound healing,^9^ cream formulations are not desirable in burn treatment and furthermore oral flutamide treatment causes adverse effects, such as fatal hepatoxicity, sexual dysfunction, anemia, and reduced muscle and bone mass.^11^

The electrospun PCL‐scaffold is here proved to deliver stable androgens (DHT) and anti‐androgens (F) or the combination of DHT plus F without burst release or any adverse effects up to 31 days in vivo. The released amount is efficient compared with the previous report of mice receiving 30 µg/day topical flutamide or 10 µg/day testosterone ointment for 6 days, while results showed that androgen antagonism promotes cutaneous wound healing.^9^ Moreover, these biomaterial scaffolds can also serve a dual purpose as a wound dressing material and dermal template for supporting dermal regeneration and repair.^8^

In our previous study, we found that there are no differences in wound healing between female WT mice and female AR knockout (ARKO) mice post‐injury.^8^ Therefore, in the present study, we only applied DHT, F, and DHT/F‐loaded scaffolds on male mice. In addition, the mice used in the validation of contorl drug delivery were castrated to minimize the endogenous androgen effect for the steroid mass spectrometry analysis to accurately measure the amount of DHT release from scaffolds.

In the in vivo burn injury castrated animal model, burst release of DHT into circulation on days 3 and 7 was observed and is likely due to the fact that topical administration of DHT scaffolds to open large wounds provided a desirable environment for drug release into the circulation. Treatment of drug‐loaded PCL scaffolds on day 7 post‐debridement was found to be most locally effective with the minimal systemic distribution. This is an important factor in the local treatment of steroids such as androgens and flutamide as systemic distribution may induce undesirable side effects on multiple tissues as previously reported.^12^

The present study confirmed that topical administration of F accelerated burn wound healing in mice as reported previously in non‐burn cutaneous wounds showing F accelerated epithelial migration.^8^, ^9^, ^16^ However, unlike cutaneous non‐burn injury wounds, local delivery of a pure, potent non‐aromatizable androgen, DHT, in cutaneous burn injury demonstrated no inhibitory effects on wound healing. This finding may be associated with the positive effects of DHT on epithelial proliferation countering with the negative effects of DHT on re‐epithelization of non‐burn skin wounds.^8^, ^9^, ^17^ By using the PCL scaffolds, re‐epithelialization was more advanced in the F scaffold‐treated mice compared with the DHT group after burn injury, comparable with previous studies showing 5α reductase inhibitor or AR inhibitor–treated mice had accelerated re‐epithelialization and faster wound gap closure.^8^, ^9^, ^17^ Interestingly, comparable epithelial migration rate was measured between DHT‐treated mice and blank control mice both in vitro and in vivo. In the present study, epithelial cell proliferation was significantly enhanced in DHT‐treated mice compared with the F‐treated mice, suggesting androgens could promote cell proliferation (i.e., keratinocytes) in a special case of major burn injury. This finding is parallel with data showing androgens to enhance the expression of keratinocyte growth factor and stimulate non‐hair follicle keratinocytes or dermal epithelial cell proliferation in mice.^4^, ^18^, ^19^, ^20^ Few other studies reported no influence of androgens^17^ or inhibit cell proliferation of keratinocytes.^8^ This controversy was probably due to previous in vitro culture systems were not specifically related to the wound environment and did not account for the possible in vivo crosstalk between different cell types such as keratinocytes and fibroblasts, in burn wound healing.

Local delivery of F from PCL scaffolds significantly increased wound collagen accumulation in mice, suggesting dermal remodeling was more rapid in anti‐androgen‐treated mice after major burn injury. The finding is consistent with previous studies where increased collagen deposition was observed during cutaneous wound healing in castrated or ARKO mice.^21^ Because the main mechanism of wound healing in rodents is by wound contraction, which relied on re‐epithelialization and matrix synthesis, studies have been suggested that androgens inhibit cutaneous wound healing by regulating keratinocytes' migration rather than proliferation.^5^ Here, under major burn injury, androgens played a positive role in keratinocytes proliferation but a negative role in keratinocytes migration and collagen deposition leading to DHT scaffold‐treated mice having slower burn wound healing rate compared with F scaffold‐treated mice.

**FIGURE 6:**
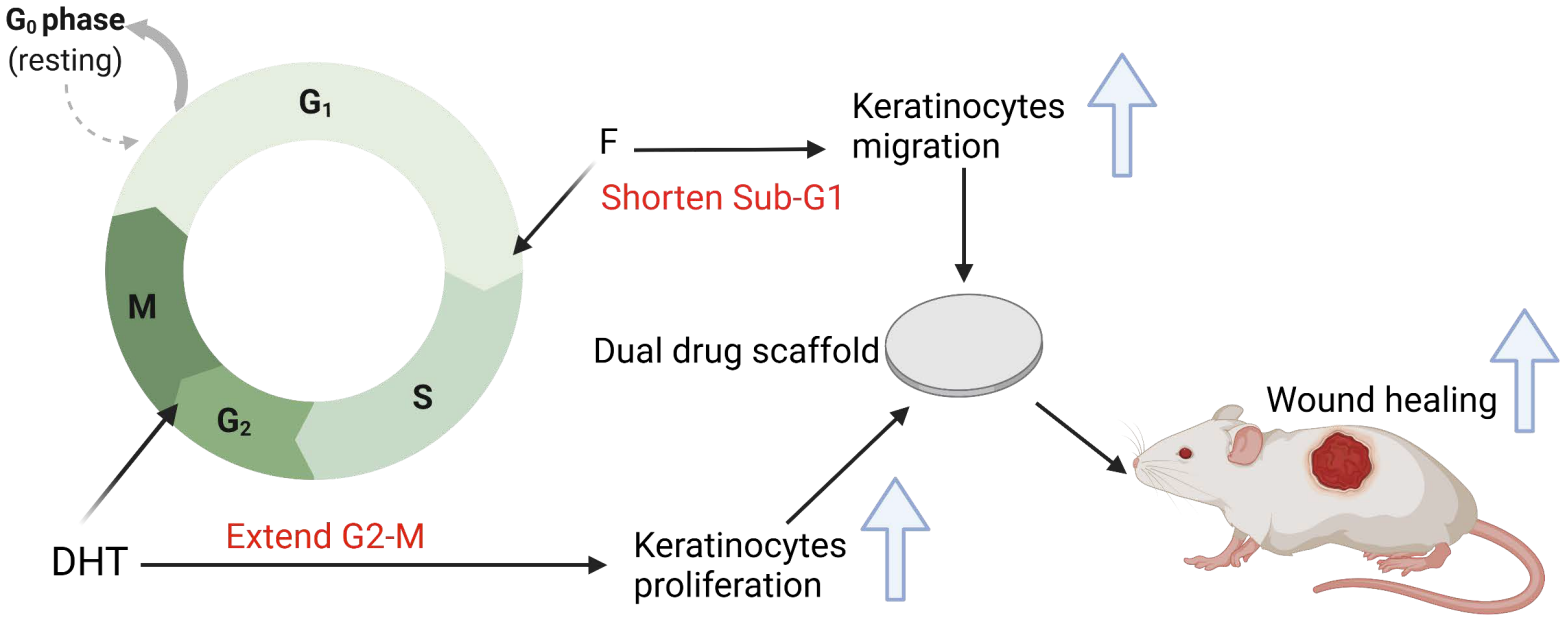
Illustration of dual‐drug scaffold accelerate burn injury wound healing via enhanced keratinocytes proliferation and migration with altered keratinocyte cell cycle

In this study, we show that combination of DHT and F within a single electrospun PCL scaffold accelerates burn injury wound healing. Histological analysis revealed that combination of DHT and F can overcome the inhibitory role of single DHT on re‐epithelialization with increased epithelial cell proliferation. As the consequence, the DHT‐F scaffold was found to further promote anagenesis, followed by enhanced collagen deposition with increased fibroblast proliferation. These findings were supported by the evidence that DF scaffold was able to enhance human keratinocytes proliferation via changing its cell cycle. The keratinocyte cell cycle analysis showed that F treatment significantly reduced the apoptotic Sub‐G1 phase, whereas the administration of DHT extended the G2‐M phase with more keratinocytes undergo mitosis. Therefore, combination of DHT and F scaffolds had the most significant effects on major burn injury wound healing as it uses their beneficial effects via androgen action on the local wound healing process (Figure 6). However, further studies are needed to explain this observed synergistic effects from pure androgen (DHT) and its inhibitor (F). Perhaps, it has an indirect effect on other tissues and the time course of effects for these two drugs differs.

To determine whether sustained controlled drug delivery can minimize adverse effects such as liver toxicity, the safety of drug loaded PCL scaffold treatments was investigated histologically and biochemically. Topical administration of DHT, F, or DF‐PCL scaffold caused no apparent systemic adverse effects, as indicated by no alterations in the androgen‐dependent organs weight and spleen, kidney, liver, teste histology, as well as markers of liver dysfunction in the serum of scaffold‐treated animals. Therefore, the combination of DHT and F PCL could serve as an improved novel approach to accelerating healing of severe cutaneous burn injury. This warrants evaluation in a well‐designed placebo‐controlled clinical trial of the translational potential of DF‐PCL scaffolds. Moreover, since the dual release of DHT/F promotes local wound healing with enhanced keratinocyte and fibroblast proliferation, it would also be applied to other kinds of wounds include full‐thickness, diabetic, or infectious wounds.

## 5 CONCLUSION

In summary, study here showed for the first‐time that local delivery of the androgen DHT in combination with its inhibitor flutamide, via electrospun PCL scaffold significantly accelerate burn injury wound healing without any systemic adverse effects. The effects of local androgen modification promoting wound healing is primarily by enhanced keratinocytes proliferation and migration with altered keratinocyte cell cycle. These exciting findings can open novel therapeutic avenues in treating major burn injury with the combination of positive effects from DHT and F mixed‐PCL scaffold.

## DISCLOSURES

Authors have no conflict of interest to declare.

## AUTHOR CONTRIBUTIONS

Huaikai Shi, David J. Handelsman, Peter K. Maitz, Mark S. Cooper, and Yiwei Wang designed the study. Huaikai Shi, Kevin H.‐Y. Tsai, Duncan Ma, Roxanne J. Parungao, Xiaosuo Wang, Reena Desai, Nicholas J. Hunt, and Yiwei Wang contributed to experiments. Huaikai Shi analyzed the data. Huaikai Shi, Nicholas J. Hunt, Yuen Yee Cheng, and Yiwei Wang wrote the paper. Hao Zhang, Ye Xu, Ulla Simanainen, Qian Tan, Mark S. Cooper, and David J. Handelsman assisted in the preparation of the manuscript. David J. Handelsman, Peter K. Maitz, and Yiwei Wang supervised the project.

## Supporting information

Fig S1‐S2

## Data Availability

The data that support the findings of this study are available on request from the corresponding author. The data are not publicly available due to privacy or ethical restrictions.

## References

[1] Jeschke MG . Post‐burn hypermetabolism: past, present and future. J Burn Care Res. 2016;37:86‐96. 26132047 10.1097/BCR.0000000000000265 PMC4691431

[2] Jeschke MG , Chinkes DL , Finnerty CC , et al. Pathophysiologic response to severe burn injury. Ann Surg. 2008;248:387‐401. 18791359 10.1097/SLA.0b013e3181856241 PMC3905467

[3] Ahn CS , Maitz PK . The true cost of burn. Burns. 2012;38:967‐974. 22795515 10.1016/j.burns.2012.05.016

[4] Ashcroft GS , Mills SJ . Androgen receptor‐mediated inhibition of cutaneous wound healing. J Clin Invest. 2002;110:615‐624. 12208862 10.1172/JCI15704 PMC151108

[5] Lai JJ , Lai KP , Chuang KH , et al. Monocyte/macrophage androgen receptor suppresses cutaneous wound healing in mice by enhancing local TNF‐alpha expression. J Clin Invest. 2009;119:3739‐3751. 19907077 10.1172/JCI39335 PMC2786793

[6] Ashcroft GS , Mills SJ , Flanders KC , et al. Role of Smad3 in the hormonal modulation of in vivo wound healing responses. Wound Repair Regen. 2003;11:468‐473. 14617288 10.1046/j.1524-475x.2003.11614.x

[7] Gilliver SC , Ruckshanthi JP , Hardman MJ , Nakayama T , Ashcroft GS . Sex dimorphism in wound healing: the roles of sex steroids and macrophage migration inhibitory factor. Endocrinology. 2008;149:5747‐5757. 18653719 10.1210/en.2008-0355

[8] Wang Y , Simanainen U , Cheer K , et al. Androgen actions in mouse wound healing: minimal in vivo effects of local antiandrogen delivery. Wound Repair Regen. 2016;24:478‐488. 26873751 10.1111/wrr.12420

[9] Toraldo G , Bhasin S , Bakhit M , et al. Topical androgen antagonism promotes cutaneous wound healing without systemic androgen deprivation by blocking β‐catenin nuclear translocation and cross‐talk with TGF‐β signaling in keratinocytes. Wound Repair Regen. 2012;20:61‐73. 22276587 10.1111/j.1524-475X.2011.00757.x PMC5461922

[10] Shi H , Lo T , Ma D , et al. Dihydrotestosterone (DHT) enhances wound healing of major burn injury by accelerating resolution of inflammation in mice. Int J Mol Sci. 2020;21:6231. 10.3390/ijms21176231 PMC7504698 32872240

[11] Boateng JS , Matthews KH , Stevens HN , Eccleston GM . Wound healing dressings and drug delivery systems: a review. J Pharm Sci. 2008;97:2892‐2923. 17963217 10.1002/jps.21210

[12] Wolverton SE . Major adverse effects from systemic drugs: defining the risks. Curr Probl Dermatol. 1995;7:6‐38.

[13] Baranoski S . Wound dressings: a myrid of challenging decisions. Home Healthcare Nurse. 2015;23:307‐317. 10.1097/00004045-200505000-00009 15891476

[14] Chong C , Wang Y , Maitz PK , Simanainen U , Li Z . An electrospun scaffold loaded with anti‐androgen receptor compound for accelerating wound healing. Burns Trauma. 2013;1:95‐101. 27574631 10.4103/2321-3868.118935 PMC4978100

[15] Harwood DT , Handelsman DJ . Development and validation of a sensitive liquid chromatography‐tandem mass spectrometry assay to simultaneously measure androgens and estrogens in serum without derivatization. Clin Chim Acta. 2009;409:78‐84. 19747904 10.1016/j.cca.2009.09.003

[16] Ashcroft GS , Greenwell‐Wild T , Horan MA , Wahl SM , Ferguson MW . Topical estrogen accelerates cutaneous wound healing in aged humans associated with an altered inflammatory response. Am J Pathol. 1999;155:1137‐1146. 10514397 10.1016/S0002-9440(10)65217-0 PMC1867002

[17] Gilliver SC , Ruckshanthi JP , Hardman MJ , Zeef LA , Ashcroft GS . 5alpha‐dihydrotestosterone (DHT) retards wound closure by inhibiting re‐epithelialization. J Pathol. 2009;217:73‐82. 18855875 10.1002/path.2444

[18] Kumtornrut C , Yamauchi T , Koike S , Aiba S , Kenshi Y . Androgens modulate keratinocyte differentiation indirectly through enhancing growth factor production from dermal fibroblasts. J Dermatol Sci. 2019;93:150‐158. 30792099 10.1016/j.jdermsci.2019.01.007

[19] Planz B , Wang Q , Kirley SD , Marburger M , McDougal WS . Regulation of keratinocyte growth factor receptor and androgen receptor in epithelial cells of the human prostate. J Urol. 2001;166:678‐683. 11458116

[20] Planz B , Wang Q , Kirley SD , Lin CW , McDougal WS . Androgen responsiveness of stromal cells of the human prostate: regulation of cell proliferation and keratinocyte growth factor by androgen. J Urol. 1998;169:1850‐1855. 10.1016/s0022-5347(01)62431-5 9783973

[21] Gilliver SC , Ruckshanthi JP , Atkinson SJ , Ashcroft GS . Androgens influence expression of matrix proteins and proteolytic factors during cutaneous wound healing. Lab Invest. 2007;87:871‐881. 17607299 10.1038/labinvest.3700627

