# Supplementary material for: Controlled dual release of dihydrotestosterone and flutamide from polycaprolactone electrospun scaffolds accelerate burn wound healing
Source: FASEB J. 2022 Apr 8;36(5):e22310. doi: 10.1096/fj.202101803R (PMC9540550; doi:10.1096/fj.202101803R)
Supplement: Supplementary file 1 — Fig S1‐S2 [file FSB2-36-0-s001.docx]

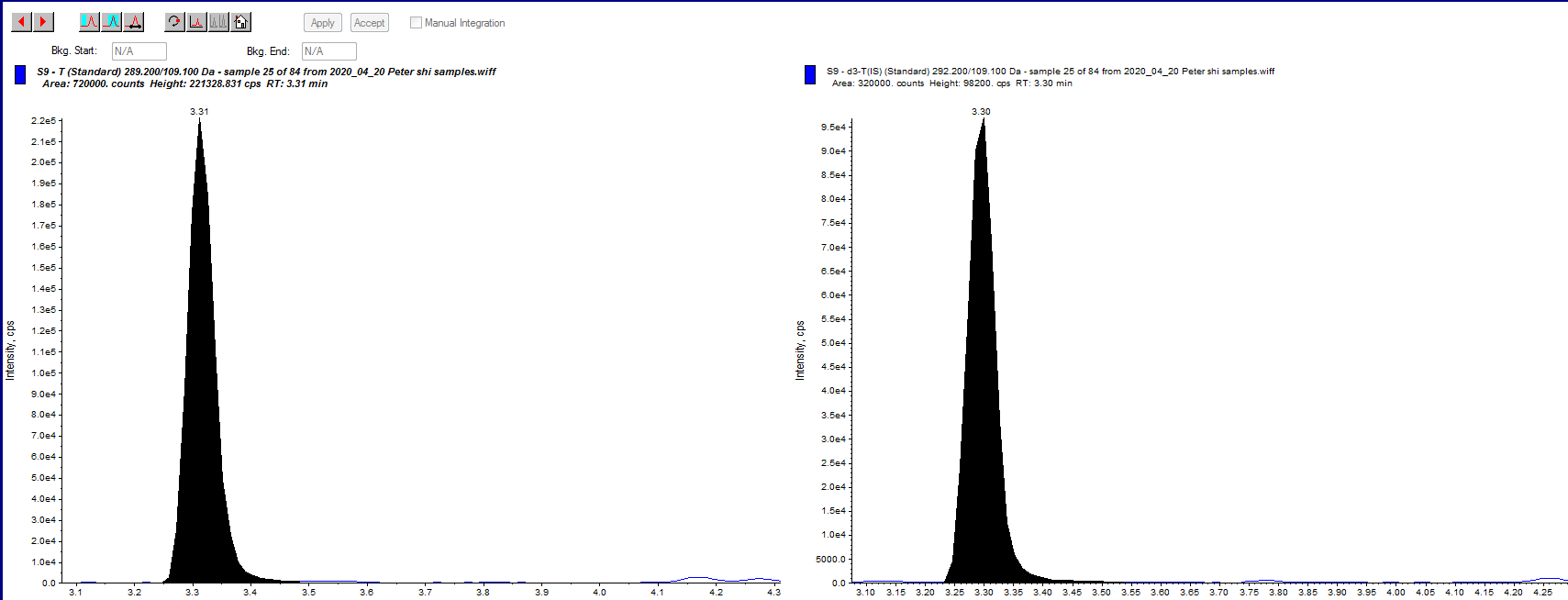


***Testosterone (T)***

***MRM: 289.2 > 109.1***

***D3-Testosterone (d3-T)***

***MRM: 292.2 > 109.1***

**(a)**


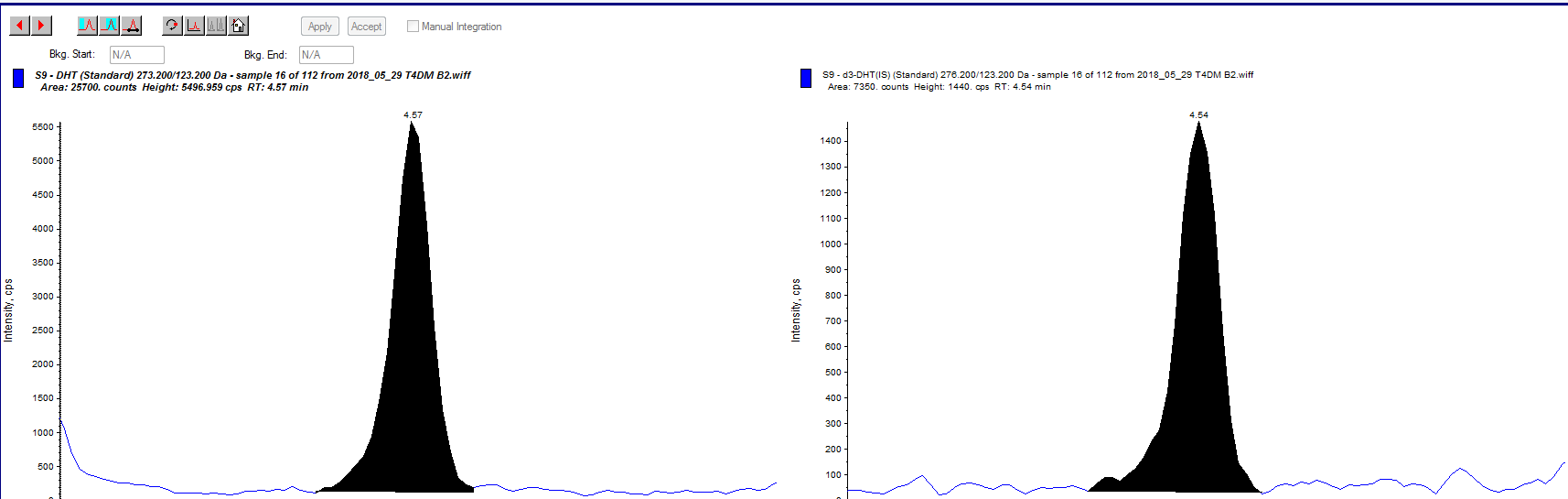


***Dihydroestosterone (DHT)***

***MRM: 273.2 > 123.2***

***D3-Dihydroestosterone (d3-DHT)***

***MRM: 276.2 > 123.2***

**(b)**

**Supplementary figure 1.** RT showed the detection of (a) T and d3-T (internal standard) at 3.28 min, (b) DHT and d3-DHT (internal standard) at 4.19 min.

**Concentration (ng/ml)**

**Supplementary figure 2.** Orchidectomy or castration significantly reduced serum testosterone and DHT concentration in mice. N=3 per group, error bar = SEM, analysed by one-way ANOVA.
